# Factors negatively affecting quality of life in geriatric patients: review

**DOI:** 10.3205/dgkh000564

**Published:** 2025-07-09

**Authors:** Krzysztof Marcinkowski, Mateusz Muras, Maciej Michalik, Tomasz Lorenc, Natalia Mikszta, Jakub Mikszta, Julia Marcinkowska, Karolina Kantor

**Affiliations:** 1 Szpital Specjalistyczny im. Ludwika Rydygiera w Krakowie, Kraków, Poland; 2 Wojewódzki Szpital Specjalistyczny nr 2 w Jastrzębiu Zdroju, Jastrzębie Zdrój, Poland; 3 Warszawski Szpital Południowy, Warszawa, Poland; 4 Samodzielny Publiczny Zakład Opieki Zdrowotnej Wojewódzki Szpital Specjalistyczny nr 3 w Rybniku, Rybnik, Poland; 5 Faculty of Medical Sciences in Katowice, Medical University of Silesia, Katowice, Poland

**Keywords:** elderly, quality of life, loss of health, mental health problems, frailty, urinary incontinence

## Abstract

**Introduction:**

The aging process is accompanied by a variety of challenges that significantly impact the quality of life and overall well-being of older adults. This study explores several key factors that negatively affect seniors, including loneliness, frailty syndrome, falls, polypharmacy, urinary incontinence, chronic pain, and malnutrition.

**Method:**

The PubMed and Google Scholar databases were searched to find scientific articles in which the terms “elderly”, and “quality of life” appear in the title, abstract, or keywords.

**Results:**

Loneliness emerges as a critical issue, contributing to mental health problems such as depression and cognitive decline. Falls, a leading cause of injury among older adults, often result in long-term physical and psychological consequences. Polypharmacy, while intended to manage multiple chronic conditions, increases the risk of adverse drug reactions and medication non-adherence. Frailty syndrome reduces a patient’s susceptibility to stressors. Urinary incontinence, often stigmatized, affects physical comfort and social interactions, reducing the dignity of those affected. Chronic pain disrupts daily functioning and exacerbates feelings of helplessness, while malnutrition undermines physical health, leading to frailty and increased susceptibility to illnesses. All of these factors have their impact on quality of life in older adults.

**Conclusions:**

The study identifies key factors negatively affecting the quality of life in older adults, including physical, psychological, and social challenges that require comprehensive and targeted interventions.

## Zusammenfassung

**Einleitung:**

Der Alterungsprozess wird von einer Vielzahl von Herausforderungen begleitet, die die Lebensqualität und das allgemeine Wohlbefinden älterer Menschen erheblich beeinträchtigen. In der Studie werden mehrere Schlüsselfaktoren untersucht, die sich negativ auf Senioren auswirken, darunter Einsamkeit, Gebrechlichkeitssyndrom, Stürze, Polypharmazie, Harninkontinenz, chronische Schmerzen und Mangelernährung.

**Methode:**

Die Datenbanken PubMed und Google Scholar wurden nach wissenschaftlichen Artikeln durchsucht, in denen die Begriffe „ältere Menschen“ und „Lebensqualität“ im Titel, in der Zusammenfassung oder in den Schlüsselwörtern vorkommen.

**Ergebnisse:**

Einsamkeit erweist sich als ein kritisches Problem, das zu psychischen Problemen wie Depressionen und kognitivem Abbau beiträgt. Stürze, eine der häufigsten Verletzungsursachen bei älteren Erwachsenen, haben oft langfristige physische und psychische Folgen. Die Polypharmazie, mit der mehrere chronische Erkrankungen behandelt werden sollen, erhöht das Risiko von unerwünschten Arzneimittelwirkungen und der Nichteinhaltung der Medikation. Das Gebrechlichkeitssyndrom verringert die Anfälligkeit der Patienten für Stressfaktoren. Harninkontinenz, die oft stigmatisiert wird, beeinträchtigt das körperliche Wohlbefinden und die sozialen Interaktionen und mindert die Würde der Betroffenen. Chronische Schmerzen beeinträchtigen das tägliche Funktionieren und verstärken das Gefühl der Hilflosigkeit, während Mangelernährung die körperliche Gesundheit untergräbt und zu Gebrechlichkeit und erhöhter Anfälligkeit für Krankheiten führt. All diese Faktoren wirken sich auf die Lebensqualität älterer Menschen aus.

**Schlussfolgerungen:**

Die Studie identifiziert Schlüsselfaktoren, die sich negativ auf die Lebensqualität älterer Erwachsener auswirken, einschließlich physischer, psychologischer und sozialer Herausforderungen, die umfassende und gezielte Interventionen erfordern.

## Introduction

The percentage of elderly people in the population is constantly increasing and, given the lengthening of the assumed lifespan, this process will intensify in the coming years [1], [2]. According to Główny Urząd Statystyczny, people aged ≥65 in 2050 will make up 31.5% of the population in Poland, and their numbers will increase by 5.1 million compared to 2014. In turn, the total number of people of retirement age could rise to as many as 10 million by 2050 [3]. The aging of the population calls for increased attention to the needs, functioning and quality of life of the elderly [4]. The incidence of diseases in the elderly is increasing significantly.

In the past, attempts have been made to define quality of life, such as: “a conscious cognitive judgment of satisfaction with one’s life” [5] or “an overall general well-being that comprises objective descriptors and subjective evaluations of physical, material, social, and emotional well-being together with the extent of personal development and purposeful activity, all weighted by a personal set of values” [6]. On the other hand, the WHO presented the concept of quality of life as “an individual’s perception of their position in life in the context of the culture and value systems in which they live and in relation to their goals, expectations, standards and concerns. It is a broadly ranging concept affected in a complex way by the person’s physical health, psychological state, level of independence, social relationships, and their relationship to salient features of their environment.” [7].

Reduced quality of life in elderly patients is largely due to functional limitations. These disorders are very common among the elderly, but they can be avoided to a certain extent, depending on the functionality of health care systems [8]. It is possible that loss of quality of life for seniors is perceived in the same way as loss of health [9]. In this article, some of the most important functional disorders that negatively affect patients’ quality of life are highlighted.

The purpose of the present article was to review and analyze the Polish and international literature on selected factors negatively affecting the quality of life of the elderly. .

## Results

### Frailty syndrome 

This is a clinical condition in which, upon exposure to a stressor, an individual’s vulnerability for heightened dependency and/or mortality increases [10]. This syndrome is common in elderly population [4]. Vitamin D deficiency, sarcopenia, malnutrition, loneliness, multidrug use, or increased oxidative stress can be blamed for the development of frailty in the elderly [11], [12]. It is characterized by a decline in the body’s physiological reserves, leading to reduced physical fitness, chronic fatigue, slowed movement, reduced daily activity and weight loss. This is the stage between full fitness and disability, which significantly affects the overall functioning and quality of life of seniors [4], [11]. Frailty syndrome promotes falls in the elderly [13], and geriatric patients are at higher risk of dementia [14]. Although no standard diagnostic tests exist for frailty syndrome, systematic attempts should be made to prevent the occurrence of the syndrome, including a comprehensive guarantee of medical care and assistance in performing daily activities [3].

### Chronic pain 

Pain, according to the International Association for Study of Pain, is defined as an unpleasant sensory and emotional experience associated with actual or potential tissue damage or is described in terms of such damage [15]. Fundamentally, pain is essentially a warning function. However, in the case of chronic pain, that is, persistent or recurrent pain lasting more than 3 months [16], which is a common experience in the elderly, is devoid of a warning function, and is only a sensation that reduces the patients’ quality of life. As for the Polish population, the most common complaints of pain are in the lower extremities and lower spine [17]. Chronic pain is associated with another factor in reduced quality of life, such as falls, increasing its risk [18]. Chronic pain, and especially the timing and location of the pain, along with other factors such as diabetes and depression, negatively affect sensory abilities, autonomy, social activities, etc. [19]. Appropriate physical exercise can reduce chronic pain and improve patients’ quality of life [20].

### Falls 

Falls are among the most common problems in modern geriatrics and have a significant impact on the quality of life of seniors. According to the WHO, about 32–42% of people over 70 years of age fall each year, which is associated with increased health care costs for the elderly and greater involvement of family and medical staff [2]. In a 2014 survey, 28.7% of older adults reported falling; an estimated 29.0 million falls caused 7.0 million injuries in the US in 2014 [21]. Falls contribute in many ways to a negative impact on patients’ quality of life [22]. Both the number of risk factors for falls and the number of falls affect the quality of life of older people [23]. It has been noted that falls are more common in women, people who live alone, people who move slowly, and those with multiple diseases [24]. Falls can negatively affect quality of life to a similar degree as chronic diseases [25]. Falls are one of the leading causes of pain, disability and premature death in seniors [2]. Also, the very phenomenon of fear of falls can negatively affect quality of life in older patients [25], [26]. Geriatric individuals who have a higher education, engage in physical exercise, and are of normal weight who have experienced falls rate their quality of life better [27]. Interventions to prevent falls can significantly improve the quality of life for geriatric patients [28]. Such interventions include screening older adults for falls, reviewing and modifying existing treatments that may affect falls, and vitamin D supplementation to improve musculoskeletal health [21].

### Urinary incontinence 

Urinary incontinence is a bothersome condition involving involuntary urine leakage [29]. It is common in the elderly, an ailment that is a nuisance to patients and those around the patient [30]. People affected by the inability to hold urine are more likely to be depressed and have a poorer perception of their health [31]. Urinary incontinence is a disorder significantly associated with quality of life, significantly affecting reduced motor activity, life satisfaction, increased pain, discomfort, anxiety and depression in older patients [32], [33].

### Malnutrition 

Malnutrition increases with age and is common in geriatric patients [34]. Malnourished patients are at greater risk because they require longer hospitalizations, more prescription drugs and are more susceptible to infections [35], such as pneumonia and wound infections . Malnutrition increases the incidence of hospitalization and risk of falls [36]. An important aspect of preventing malnutrition is the documentation of body weight and its changes [37]. Malnutrition reduces quality of life in geriatric patients [34]. Protein-energy malnutrition is associated with increased mortality [38]. Adequate nutrient supplementation of geriatric patients during hospitalization can contribute to a reduced risk of mortality and complications [39].

### Polypharmacy 

As patients age, the number of medications they take increases. Polypharmacy, or multidrug use, is defined as taking a minimum of five different medications per day [40]. Polypharmacy is a common phenomenon in the elderly, the risk of which is increased by the lack of or little coordination of patient care [41]. The risk of multidrug use increases with patient age, which also correlates with the number of comorbidities, especially among oncology patients, or those under palliative care [42]. Polypharmacy, due to the risk of side effects and drug interactions, can pose a real risk to the patient; hence, pharmacological management requires particular caution. Studies have shown an association between the use of polypharmacy and negative effects on renal excretory function [43], increased risk of falls, especially during the use of fall risk-increasing drugs (FRIDS) and bone-related medication (BRMs) [42], cognitive function, especially during long-term poly-pharmacy [44], hemorrhagic and thrombotic events, etc. [42]. Due to the many comorbidities in elderly patients, the use of multiple drugs is sometimes necessary. It is imperative to consider the possible complications of such intensified therapy and proceed in a manner which maximizes the therapeutic benefit to the patient.

### Loneliness 

At advanced ages, patients are more likely to suffer from morbidity, widowhood, restricted income and mobility limitations [45]. All of these factors have a role in increasing social isolation. Although social isolation is seen as an objective condition that limits interpersonal contact, it is not the same as loneliness, which is a subjective deficiency of interpersonal relationships; they are related concepts and are associated with negative consequences for the quality of life of older people [46]. Loneliness in older people is seen as a risk factor for depression and anxiety [47], and living alone is a risk factor with an increased mortality rate [48]. Loneliness among the elderly is linked to limited physical fitness, multimorbidity and the patient’s perception of illness. [49]. Even brief episodes of isolation can significantly affect the psychological well-being of the elderly [50].

Elderly patients experience an increased need for social contacts to support and assist them [51]. Activities which provide social and emotional attention can yield tangible benefits in improving the quality of life of elderly patients. The absence of loneliness can promote a good quality of life and help patients adapt to their disease state and related treatment [46]. 

### Increased susceptibility to infections due to senescence 

In general, all cells are affected by cellular aging processes. If, for example, the number of T lymphocytes decreases, this results in an increased susceptibility to infection. Age-associated diseases such as neurodegenerative or cardiovascular diseases, type 2 diabetes mellitus, tumor diseases, but also general frailty are directly associated with the impairment of the immune system and with changes in the gut microbiome [52], [53], [54]. An important feature of senescence is the transformation of certain body cells into a “senescence-associated phenotype”, which is associated with the production of inflammatory substances [55]. In the context of the aging process, the chronic exposure of the body to these inflammatory substances (interleukins, acute phase proteins, etc.) is referred to as “inflammaging” [56].

### Visual impairment 

Reduced visual ability results from the decreasing spectral transmissibility of the eye with increasing age with a reduction in the production of the sleep hormone melatonin, leading to sleep disorders and the restriction of anti-inflammatory regulatory processes [57], [58]. The light requirements of older people therefore increase not only for vision, but also for the function of circadian synchronization and the immune response [57].

## Conclusions

The study highlights several risk factors that significantly diminish the quality of life in older patients, including loneliness, falls, polypharmacy, urinary incontinence, frailty syndrome, chronic pain and malnutrition. These factors are often interrelated, exacerbating their impact on physical and mental well-being. Recognizing these risks is crucial in providing appropriate care and support, as inadequate management can lead to a decline in overall health and independence. Proper care for older patients requires a comprehensive understanding of these challenges to mitigate their effects and safeguard the dignity and quality of life of this vulnerable population.

## Notes

### Competing interests

The authors declare that they have no competing interests.

### Funding

None. 

### Authors’ ORCIDs

- Marcinkowski K: https://orcid.org/0009-0002-5759-7285
- Muras M: https://orcid.org/0009-0005-1392-3773
- Michalik M: https://orcid.org/0009-0009-7799-7252
- Lorenc T: https://orcid.org/0009-0008-9902-469X
- Mikszta N: https://orcid.org/0009-0003-8444-7650
- Mikszta J: https://orcid.org/0009-0000-4194-9915
- Marcinkowska J: https://orcid.org/0009-0005-7006-4303
- Kantor K: https://orcid.org/0009-0005-0484-2883
